# Correlation between blood urea nitrogen/albumin levels and 30-day all-cause mortality in critically Ill patients with heart failure: a retrospective cohort study and predictive model development based on machine learning

**DOI:** 10.3389/fcvm.2025.1600640

**Published:** 2025-09-05

**Authors:** Wen-Ting Sun, Pei-Feng Chang, Wen-Hui Liu, Bo Liu, Wu-Jiao Wang, Xing-Yue Wang, Hong Su, Yi Tang, Tian-li Li

**Affiliations:** ^1^ Department of Integrated Cardiology, China-Japan Friendship Hospital, Beijing, China; ^2^ Beijing University of Chinese Medicine, Beijing, China; ^3^ Department of Cardiovascular Medicine, Dongzhimen Hospital, Beijing, China; ^4^ Department of Pain Management, China-Japan Friendship Hospital, Beijing, China; ^5^ Department of Neurology, Hefei Hospital Affiliated to Anhui Medical University (The Second People's Hospital of Hefei), Hefei, China

**Keywords:** BAR index, heart failure, ICU, MIMIC-IV database, 30-day mortality

## Abstract

**Objective:**

The aim of this study was to investigate the correlation between blood urea nitrogen-albumin index (BAR) and 30-day and one-year all-cause mortality in patients with heart failure admitted to the intensive care unit (ICU).

**Method:**

This is a retrospective cohort study with data from two non-overlapping datasets from the Medical Information Marketplace in Intensive Care (MIMIC), where MIMIC-IV was used for training and MIMIC-III for external validation. Risk ratios (HR) and 95% confidence intervals (CI) between the BAR index and all-cause mortality were assessed using Cox proportional risk regression and Kaplan–Meier curves. Restricted cubic spline regression modeling was used to assess potential nonlinear relationships between BAR indices and outcome indicators. Nine machine learning (ML) algorithms were used to build predictive models, and, in addition, the Shapley additive interpretation (SHAP) method was used to determine feature importance.

**Result:**

This study included 2,470 critically ill heart failure patients. Multivariate Cox regression analysis revealed that the risk of all-cause mortality was significantly higher at both 30 and 365 days for patients in the highest quartile of the BAR index. Kaplan–Meier analyses indicated that the cumulative incidence of mortality increased with higher quartiles of the BAR ratio. Additionally, multivariate restricted cubic spline regression showed a nonlinear increase in death risk at 30 and 365 days with higher BAR index values. Subgroup analyses confirmed consistent effect sizes and stability across groups. Among the nine models, XGBoost performs the best, with an AUC value of 0.894 [95% confidence interval (CI): 0.85–0.93] in the internal validation dataset and 0.924 [95% confidence interval (CI): 0.88–0.96]. The model demonstrated the best predictive performance in terms of discrimination and clinical application.

**Conclusion:**

We found that higher BAR levels were significantly associated with a higher risk of 30- and 365-day all-cause mortality in critically ill patients with heart failure.

## Background

Cardiovascular disease has been the number one threat to global health over the past few decades, with approximately 20.8 million people dying from cardiovascular disease (CVD) every year up to 2023, accounting for approximately one-third of all global deaths (1). Heart failure (HF) is a clinical syndrome characterized by structural and functional damage to the heart and is the end stage of cardiovascular disease. Heart failure has long been a major global health challenge, especially as the prevalence of HF continues to rise with the aging population and the increasing burden of comorbidities such as hypertension, diabetes, and obesity, which not only severely reduces the quality of life of patients but also imposes a huge economic burden worldwide. Patients with heart failure often have a poor prognosis, with a five-year survival rate similar to that of malignant tumors, and it is important to actively develop predictive models for patients with heart failure to improve their prognosis.

HF is the end stage of many cardiovascular diseases, and the reduction of effective circulating blood volume and insufficient organ perfusion are important pathophysiologic mechanisms of heart failure (2). The BAR index introduced by blood urea nitrogen and albumin is a valuable biomarker discovered in recent years. Although BUN is less sensitive to renal insufficiency than serum creatinine, studies are confirming that increased BUN is associated with poor prognosis in patients with heart failure (3). The kidneys have a close relationship with the heart, and in patients with chronic heart failure, the compensatory effects of the kidneys may keep BUN low, but with heart failure loss of compensation, there is insufficient effective circulating blood volume, which leads to the secretion of a variety of neurohormones, resulting in a further reduction in renal perfusion when high levels of BUN may herald more severe heart failure. Reduced levels of albumin (ALB), an abundant soluble protein component of the circulatory system, are thought to be primarily associated with cachexia, renal insufficiency, hepatic dysfunction, and inflammation. Previous studies have shown that hypoalbuminemia is an independent predictor of poor prognosis in heart failure (4). Metabolic imbalance, inflammatory response, oxidative stress, and endothelial dysfunction are important features of heart failure, and BAR, as a combination of these two hematological indices, reflects the multiple pathophysiological processes of cardiovascular disease. Therefore, active research on the BAR index and the outcome and prognosis of heart failure is necessary to help provide better insights into the clinical management of cardiovascular disease.

Previous studies have shown that higher BAR indices are associated with an increased risk of adverse outcomes in patients with myocardial infarction (5), acute coronary syndromes (6), and after cardiac surgery. However, studies on the association between BAR index and critically ill patients with heart failure are lacking. This study investigated the correlation between the BAR index and all-cause mortality in heart failure patients admitted to the intensive care unit (ICU). The results of the study may help to explore new strategies for early identification and improvement of prognosis in critically ill patients with heart failure and provide important new insights into the function of BAR in predicting patient prognosis.

## Method

### Data source

The data used to construct the model came from the Medical Information Mart for Intensive Care IV (MIMIC-IV, version: v2.2) (7) which contains clinical information on 431,231 hospital admissions for 299,712 patients admitted to Beth Israel Deaconess Medical Center from 2008 to 2020. We also performed external validation using a subset of the MIMIC-III database (8), which included 26,836 admissions for 23,692 patients between 2001 and 2008, and there was no overlap with patients with MIMIC-IV. The MIMIC-IV database details information on patient demographics, laboratory tests, medications, vital signs, surgeries, disease diagnoses, and follow-up survival status. In order to protect patient privacy, all personal information is de-identified and a random code is used instead of patient identification, so we do not require the patient's informed consent and ethical approval. To access the database, author Yi Tang completed the Collaborative Institutional Training Initiative (CITI) course and passed the Conflict of Interest and Data or Sample Study Only exams (ID: 13870584).

### Inclusion and exclusion criteria

The study included patients with heart failure who were hospitalized for the first time and admitted to the ICU for the first time.

The inclusion criteria were as follows:

(1) Age ≥18 years.
(2) Meeting the diagnostic criteria for heart failure. In this study, heart failure and other comorbidities were diagnosed using the International Classification of Diseases, Ninth Revision (ICD-9) and Tenth Revision (ICD-10) codes (I5083, I5082, I5084, I5089,I509,40201,40291,40491,40492, 4280,4281,42820, 42821,42822,42823, 42830,42831,42832,42833, 42840,42841, 42842, 42843,4289, I5020, I5021, I5022, I5023, I5030, I5031, I5032, I5033, I5040, I5041, I5042, I5043, I50810, I50811, I50812, I50813, I50814).

The exclusion criteria were as follows:

(1) Patients with ICU hospitalization time less than 24 h.
(2) Lack of blood urea nitrogen and serum albumin in laboratory tests.
(3) For patients with multiple ICU admissions, only data from the first hospitalization were included.
(4) Patients with direct or indirect causes of abnormal release of blood urea nitrogen and albumin, including hepatitis, cirrhosis, and malignancy, were excluded from the study.

### Data collection and definitions

Structured Query Language (SQL) in PostgreSQL was used to extract data from both databases for patients admitted to the ICU within the previous 24 h. The variables extracted for this study were (1) demographics: age, gender, weight; (2) comorbidities: acute myocardial infarction (AMI), hypertension (HTN), atrial fibrillation (AF), coronary heart disease (IHD), diabetes mellitus (DM), chronic obstructive pulmonary disease (COPD), stroke (CVA), pneumonia (PNA), acute kidney injury (AKI), chronic kidney disease (CKD), hyperlipidemia (HLD). (3) Vital signs: systolic blood pressure (SBP), diastolic blood pressure (DBP), mean blood pressure (MBP), heart rate (HR), respiratory rate (RR), oxygen saturation (SPO2), temperature; (4) Drugs: angiotensin-converting enzyme inhibitors/angiotensin receptor blockers (ACEI/ARB), diuretics, statins, antiplatelet agents, Anticoagulants; (5) Laboratory data: red blood cells (RBC), red blood cell distribution width (RDW), hematocrit (Hct), hemoglobin (Hb), platelets (PLT), white blood cells (WBC), glucose, blood urea nitrogen (BUN), creatinine, neutrophil, lymphocytes, monocytes, lactic acid (Lac), blood urea nitrogen (BUN), albumin (ALB); (6): scoring data: Oxford Acute Severity of Illness Score (OASIS), Simplified Acute Physiology Score (SAPSII), Sequential Organ Failure Assessment (SOFA), Acute Physiology and Chronic Health Score (APSIII), Acute Physiology and Chronic Health Score (APACHEII). (7): Baseline treatment: Invasive mechanical ventilation (IMV).

All laboratory indices extracted from the MIMIC-IV (2.2) database were taken from each measurement made after the patient's admission to the ICU, and the worst of these values was taken. The blood urea nitrogen-albumin ratio (BAR) was defined as an index calculated using the following formula: BAR = blood urea nitrogen/albumin. Missing values for laboratory indicators are common in the MIMIC-IV database. To minimize bias due to sample exclusion, we calculated the percentage of missing values for each continuous variable. To avoid bias, variables with missing values exceeding 20% were excluded. Variables with missing values between 5% and 10% were treated using multiple imputations, and variables with missing values less than 5% were treated using mean imputation. Outliers were filtered using the BoxPlot method.

### Outcome measures

The primary study endpoints were 30 and 365-day mortality after ICU admission, and the secondary study endpoints were in-hospital and ICU all-cause mortality.

### Statistical analysis

Categorical variables were assessed using Fisher's exact probability method or chi-square test and expressed as counts (percentages). Continuous variables were expressed as interquartile ranges, and medians were tested using the Wilcoxon rank sum test. Multicollinearity was checked using Variance Inflation Factor (VIF), and variables with VIF above 5 were deleted (9, 10). Patients were categorized into 4 groups based on the quartiles of the BAR index, with the lowest quartile as the reference group. Kaplan–Meier survival analysis was used to assess the incidence of 30-day and one-year mortality events between groups based on different BAR levels, followed by a log-rank test to assess the significance of the differences between groups and a log Rank test to compare the two curves. Restricted cubic spline (RCS) curves were used to explore potential nonlinear relationships between the BAR Index and 30-day and one-year mortality rates and to create a threshold effects model to identify inflection points in the BAR Index. Univariate and multivariate Cox regression analyses were conducted using Boruta's algorithm to characterize the screened variables and to test for trends, adjusting for several confounding variables. (Model 1: includes only the BAR index without any adjustment; Model 2 is adjusted for demographic variables such as age, gender, weight, etc.; Model 3: Combines important characteristic variables based on clinical expertise and screening from Boruta and Random Forest algorithms, including age, weight, PNA, AKI, PLT, RDW, neutrophils, HCT, blood urea nitrogen, lactate, lymphocytes, monocytes, diuretics, APSIII, SAPAII, and OASIS. Finally, we also performed subgroup analyses including sex, age, comorbidities [acute kidney injury (AKI), coronary heart disease (CKD), type 2 diabetes mellitus, diuretics, and mechanical ventilation], and assessed the *P* of the interaction using the log-likelihood ratio test. Statistical analyses for this study were performed using Python (version 3.9.12), SPSS (version 25.0), and DecisionLnc1.0 (11) software.

### The establishment and validation of predictive models

We internally validated the MIMIC-IV database by dividing the training and test sets in 7:3. Subsequently, we validated the model externally using MIMIC-III. By combining internal and external validation, we were able to comprehensively assess the performance and generalization ability of the constructed models. In this study, nine machine learning algorithms, namely Extreme Gradient Boosting (XGBoost), Support Vector Machine (SVM), Adaptive Boosting (Adaboost), Light Gradient Boosting Machine (LGB), K-Nearest Neighbor Classifier (KNNC), Decision Tree (DT), Random Forest (RF), Gradient Boosting Based (CatBoost), and Gradient Boosting Tree (GBDT), are used to construct the prediction models. In order to optimize the overall performance of the model, we implemented feature selection during the modeling process to reduce the complexity of the model and enhance generalization.

The essence of the algorithm is based on two concepts: “shadow features” and “binomial distribution”. Boruta generates a set of copies of features, called shaded features, from the original dataset. Elements were considered significant and retained if their *Z*-scores exceeded the maximum possible *Z*-score for the shaded elements, indicating that they had the largest matching effect (ratio or risk ratio) among the set of biomarkers; otherwise, they were excluded (12). The maximum area under the curve (AUC) of the subject's work characteristics (ROC) was selected as the optimal model during the parameter tuning process. The performance of the predictive model was evaluated by AUC, sensitivity, specificity, recall, F1 score, accuracy and recall of the ROC curve. In addition, decision curve analysis (DCA) and calibration curves were plotted to assess the net clinical benefit. The interpretability of the final predictive model was explored using the Shapley summation and interpretation (SHAP) method (13).

## Results

### Baseline demographic and clinical characteristics

**Figure 1:**
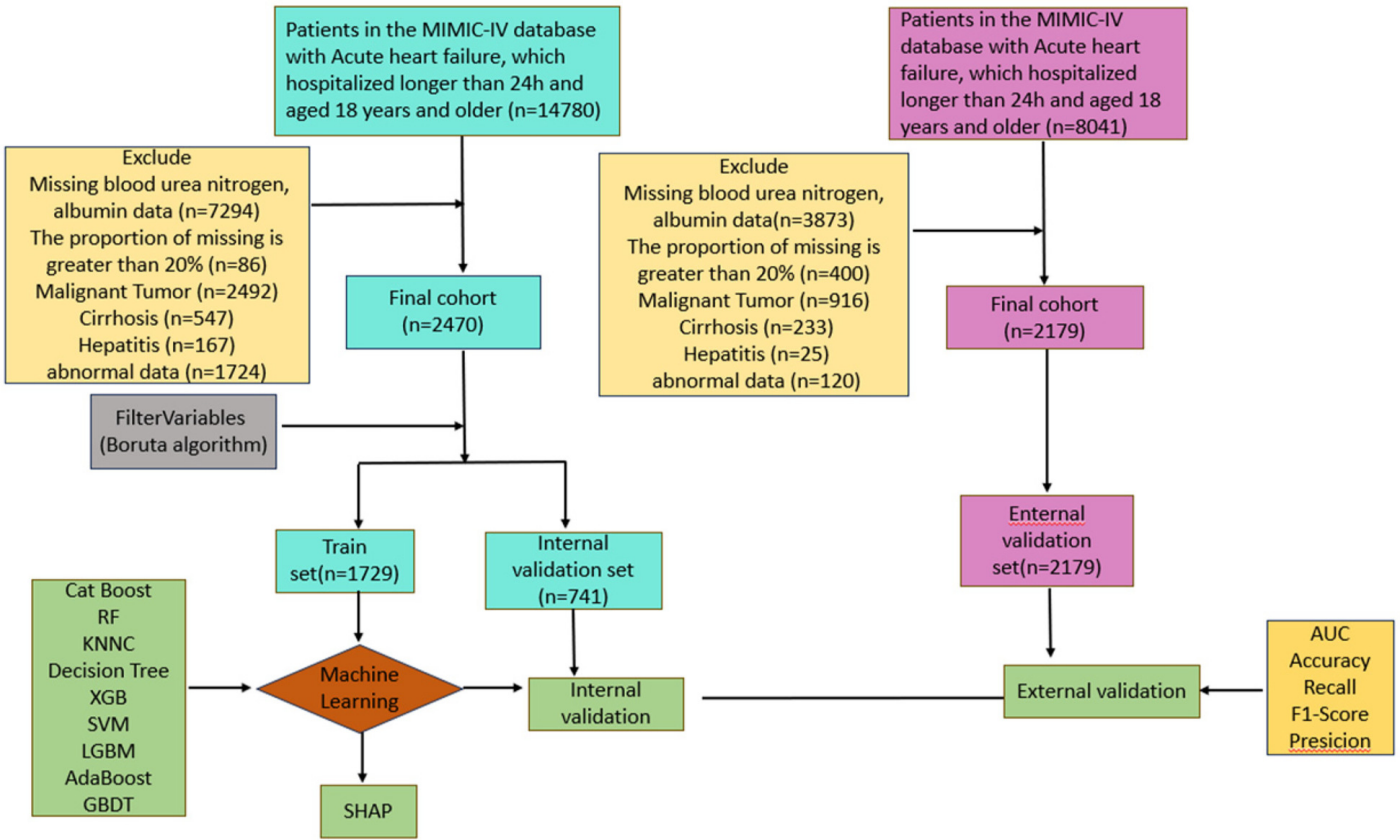
Study flow diagram depicting exclusion criteria and outcomes.

As shown in Figure 1, After screening, the study included 2470 patients from the MIMIC-IV database (mean age 72.06 years, 57.81% male) and 2179 patients from the MIMIC-III database (mean age 74.36 years, 45.30% male). Table 1 summarizes the comparison of baseline characteristics, vital signs, severity scores, comorbidities, medications used, and laboratory indices between nonsurvivors and survivors at 30 days. The comparison between the survival (2108,85.3%) and non-survival (362, 14.7%) groups showed that, in the non-survivor group, age, weight, RR, SPO2, WBC, RDW, Hb, Hct, Albumin, Urea Nitrogen, Lactic acid, Glucose, Creatinine, Neutrophil, SOFA, SAPS II, OASIS, APSIII, APACHEII, PNA, HLD, AKI, CKD, ACEI/ARB, statins, diuretics compared to survivors significantly. In addition, we divided heart failure into heart failure with reduced ejection fraction (HFrEF), heart failure with preserved ejection fraction (HFpEF), and heart failure with intermediate ejection fraction (HFmrEF) according to the ejection fraction LVEF, and we found that HFmrEF had the highest number of people, and although the proportion of HFmrEF was slightly higher in the death group than in the survival group, this difference was not statistically significant (*p* = 0.178). Among patients receiving medications, we found that mortality was lower in patients using ACEI/ARBs, statins, and diuretics, and the use of these medications was significantly higher in the survivor group than in the fatal group, suggesting that these medications may be associated with lower mortality. However, the relationship between anticoagulants and antiplatelet agents and mortality was not significant, and further studies are needed to confirm their effects.

**Table 1:** Baseline characteristics between survivors and non-survivors.

Characteristics	MIMIC-IV (*N* = 2,470)			MIMIC-III (*N* = 2,179)		
	Survivor *N* =2,108	Non-survivor *N* = 362	*P*	Survivor *N* =2,026	Non-Survivor *N* =153	*P*
BAR	13.18 ± 8.15	20.58 ± 10.85	<0.001	13.40 ± 8.33	20.81 ± 11.43	<0.001
Demographic						
Age, year	71.19 ± 13.62	77.10 ± 10.44	**<0** **.** **001**	73.80 ± 12.69	81.73 ± 9.89	**<0** **.** **001**
Weight	84.19 ± 20.96	79.19 ± 21.93	**<0** **.** **001**	85.02 ± 25.42	82.61 ± 23.89	**0** **.** **009**
Gender, *n* (*p* %)						
F	65 (48.87%)	1,440 (44.97%)	0.376	900 (44.42%)	65 (42.48%)	0.027
M	68 (51.13%)	1,762 (55.03%)		1,126 (55.58%)	88 (57.52%)	
Vital signs						
HR, beats/min	89.19 ± 20.16	90.41 ± 20.84	0.302	87.79 ± 18.71	91.84 ± 19.77	**0** **.** **045**
NBPS, mmHg	118.51 ± 23.53	117.44 ± 24.35	0.440	121.48 ± 23.47	113.68 ± 25.37	**<0** **.** **001**
NBPD, mmHg	71.48 ± 150.91	66.70 ± 18.92	0.166	67.97 ± 152.13	62.86 ± 19.04	0.310
NBPM, mmHg	80.69 ± 17.81	82.35 ± 48.91	0.525	77.86 ± 17.28	74.16 ± 19.08	**0** **.** **019**
RR, times/min	19.56 ± 6.44	20.86 ± 6.26	**<0** **.** **001**	18.27 ± 5.71	20.84 ± 6.87	**<0** **.** **001**
SPO2,%	96.72 ± 4.05	95.83 ± 4.79	**<0** **.** **001**	96.83 ± 4.38	96.02 ± 5.51	0.250
Temperature, °F	98.04 ± 3.76	98.10 ± 1.39	0.572	97.64 ± 4.64	97.46 ± 1.74	**<0** **.** **001**
Laboratory tests						
RBC, 10^9^/L	2.92 ± 0.64	2.87 ± 0.66	0.137	3.13 ± 0.64	3.03 ± 0.75	**0** **.** **032**
WBC, 10^9^/L	18.32 ± 7.75	20.03 ± 9.73	**0** **.** **002**	7.51 ± 2.97	9.82 ± 6.30	**<0** **.** **001**
PLT, 10^9^/L	143.94 ± 61.47	141.39 ± 70.93	0.520	171.89 ± 82.84	146.94 ± 84.78	**<0** **.** **001**
RDW, (%)	16.07 ± 2.20	17.29 ± 2.28	**<0** **.** **001**	15.88 ± 2.11	16.94 ± 2.16	**<0** **.** **001**
Hb, g/dl	8.68 ± 1.87	8.43 ± 1.85	**0** **.** **020**	9.31 ± 1.83	8.90 ± 1.93	0.012
Hct, mg/dl	37.31 ± 5.96	35.72 ± 6.06	**<0** **.** **001**	35.62 ± 4.85	35.98 ± 5.01	0.414
Urea Nitrogen, mg/dl	42.80 ± 24.10	61.85 ± 30.01	**<0** **.** **001**	38.64 ± 20.49	52.94 ± 24.76	**<0** **.** **001**
Albumin (g/dl)	3.38 ± 0.57	3.10 ± 0.57	**<0** **.** **001**	3.04 ± 0.56	2.69 ± 0.61	**<0** **.** **001**
Lactic acid	2.70 ± 1.48	3.19 ± 1.78	**<0** **.** **001**	2.53 ± 1.80	5.49 ± 4.73	**<0** **.** **001**
Glucose (mg/dl)	198.92 ± 69.53	225.30 ± 72.90	**<0** **.** **001**	176.55 ± 58.38	191.14 ± 63.61	**<0** **.** **001**
Creatinine (mg/dl)	1.27 ± 0.61	1.44 ± 0.66	**<0** **.** **001**	1.65 ± 1.01	2.37 ± 1.37	**<0** **.** **001**
Monocyte (mg/dl)	6.64 ± 3.35	7.02 ± 3.43	0.054	5.66 ± 3.40	5.86 ± 4.31	0.925
Neutrophil (mg/dl)	81.89 ± 7.92	85.88 ± 7.12	**<0** **.** **001**	82.90 ± 9.68	85.17 ± 12.15	**<0** **.** **001**
Severity scores						
SOFA	5.42 ± 3.02	6.65 ± 3.56	**<0** **.** **001**	4.55 ± 2.75	6.83 ± 3.65	**<0** **.** **001**
APSIII	46.79 ± 17.80	56.98 ± 19.63	**<0** **.** **001**	44.88 ± 17.13	63.56 ± 25.29	**<0** **.** **001**
SAPSII	39 ± 12.14	46.10 ± 13.14	**<0** **.** **001**	37.75 ± 11.73	49.92 ± 15.63	**<0** **.** **001**
OASIS	32.77 ± 8.14	37.13 ± 8.64	**<0** **.** **001**	32.58 ± 8.25	39.65 ± 8.72	**<0** **.** **001**
APACHEII	13.15 ± 3.14	13.58 ± 2.89	0.125	11.24 ± 3.24	13.47 ± 2.18	0.145
Comorbidities						
HF			0.178			NA
HFmrEF	856 (42.50%)	190 (47.27%)				
HFpEF	620 (30.30%)	119 (25.76%)				
HFrEF	560 (27.20%)	123 (26.97%)				
AF	38 (28.57%)	763 (23.83%)	0.210	927 (45.76%)	92 (60.13%)	0.002
HTN	34 (25.56%)	1,004 (31.36%)	0.158	982 (48.47%)	59 (38.56%)	0.057
PNA	45 (33.83%)	599 (18.71%)	**<0** **.** **001**	742 (36.62%)	82 (53.59%)	**<0** **.** **001**
CVA	16 (12.03%)	354 (11.06%)	0.726	183 (9.03%)	9 (5.88%)	0.407
T2DM	48 (36.09%)	1,202 (37.54%)	0.735	851 (42.00%)	44 (28.76%)	0.006
T1DM	1 (0.75%)	27 (0.84%)	0.910	25 (1.23%)	2 (1.31%)	0.785
HLD	60 (45.11%)	1,729 (54%)	**0** **.** **044**	727 (35.88%)	44 (28.76%)	0.019
MI	16 (12.03%)	468 (14.62%)	0.407	171 (8.44%)	13 (8.50%)	0.782
CAD	76 (57.14%)	2,018 (63.02%)	0.169	78 (60.34%)	1,978 (66.78%)	0.134
COPD	24 (18.05%)	702 (21.92%)	0.288	28 (19.34%)	698 (20.56%)	0.32
AKI	1,040 (49.34%)	252 (69.61%)	**<0** **.** **001**	767 (37.86%)	98 (64.05%)	**<0** **.** **001**
CKD	640 (30.36%)	145 (40.06%)	**<0** **.** **001**	521 (25.72%)	42 (27.45%)	0.814
Drugs						
ACEI-ARB			**<0** **.** **001**	1,321 (65.20%)	50 (32.68%)	**<0** **.** **001**
No	1,411 (66.94%)	285 (78.73%)		705 (34.81%)	103 (67.32%)	
Yes	697 (33.06%)	77 (21.27%)		1,320 (65.19%)	50 (32.68%)	
Stains	462 (21.92%)	57 (15.75%)	**0** **.** **008**	1,598 (78.87%)	98 (64.05%)	**<0** **.** **001**
No	1,646 (78.08%)	305 (84.25%)		428 (21.14%)	55 (35.95%)	
Yes	462 (21.92%)	57 (15.75%)		1,597 (78.86%)	98 (64.05%)	
Diuretics	886 (42.03%)	72 (19.89%)	**<0** **.** **001**	1,856 (91.61%)	137 (89.54%)	0.617
No	1,222 (57.97%)	290 (80.11%)		170 (8.40%)	16 (10.46%)	
Yes	886 (42.03%)	72 (19.89%)		1,855 (91.60%)	137 (89.54%)	
Anticoagulants	1,963 (93.12%)	334 (92.27%)	0.555	1,879 (92.74%)	143 (93.46%)	0.851
No	145 (6.88%)	28 (7.73%)		147 (7.26%)	10 (6.54%)	
Yes	1,963.00 (93.12%)	334 (92.27%)		1,878 (92.74%)	143 (93.46%)	
Antiplatelet	488 (23.15%)	82 (22.65%)	0.835	1,155 (57.01%)	66 (43.14%)	**<0** **.** **001**
No	1,620 (76.85%)	280 (77.35%)		871 (43.01%)	87 (56.86%)	
Yes	488 (23.15%)	82 (22.65%)		1,154 (56.99%)	66 (43.14%)	

Bold text indicates statistical significance.

WBC, white blood cell; RDW, red cell distribution width; BUN, blood urea nitrogen; RBC red blood cell; HR, heart rate; SpO2, oxyhemoglobin saturation; RR, respiratory rate; PLT, platelet; RDW, red cell distribution Width; Hct, hematocrit; Hb, hemoglobin; SBP, systolic blood pressure; DBP, diastolic pressure; CKD, chronic kidney disease; T1DM, diabetes I type 1 diabetes; T2DM, diabetes II type 2 diabetes; COPD, chronic obstructive pulmonary disease; CAD, coronary artery disease; AKI, acute kidney injury; AF, atrial fibrillation; PNA, pneumonia; CVA, cerebrovascular accident; HLD, hyperlipemia; SOFA, sequential organ failure assessment; APSIII, acute physiology score III; SAPSII, simplified acute physiology score II; OASIS, oxford acute severity of illness score; APACHEII, acute physiology and chronic health evaluation II score; ICU, intensive care unit; MI, myocardial infarction; HFrEF, heart failure with reduced ejection fraction; HFpEF, heart failure with preserved ejection fraction; HFmrEF, heart failure with mid-range ejection fraction; Data are *n*/*N* (%) or mean ± standard deviation.

In Table 2, the hospital, ICU, 30-day, and 365-day mortality rates of the patients were 3.52%, 10.97%, 14.66%, and 15.3%, respectively. Patients were categorized into 4 groups based on the quartiles of the BAR index: quartile 1 (1.33 ≤ BAR < 6.58), quartile 2 (6.58 ≤ BAR < 9.39), and quartile 3 (9.39 ≤ BAR ≤ 13.94) quartile 3 (13.94 ≤ BAR ≤ 59.41), with the first 3 groups consisting of 617 individuals and the fourth group consisting of 619 individuals. Patients in quartile 4 exhibited higher weight, age, heart rate, blood pressure, respiratory rate, neutrophil count, white blood cell count, blood urea nitrogen, glucose, monocyte count, creatinine, lactate, SOFA score, mechanical ventilation, and proportion of statin use, as well as lower levels of albumin, erythrocytes, platelet count, hemoglobin, hematocrit, and SPO2.

**Table 2:** Patient demographics and baseline characteristics.

Variables	Overall *N* = 2,470	Q1 *N* = 617	Q2 *N* = 617	Q3 *N* = 617	Q4 *N* = 619	*p*
BAR	14.26 ± 8.99	5.75 ± 1.24	9.56 ± 1.12	14.64 ± 1.96	27.05 ± 7.61	**<0** **.** **001**
Demographic						
Weight	83.45 ± 21.17	82.84 ± 20.54	82.84 ± 20.75	83.75 ± 21.67	84.38 ± 21.72	0.514
Age, year	72.06 ± 13.37	65.85 ± 14.71	72.45 ± 12.47	74.65 ± 12.56	75.28 ± 11.40	**<0** **.** **001**
Male, *n* (%)	1,428 (57.81%)	350 (56.73%)	347 (56.24%)	344 (55.75%)	387 (62.52%)	0.055
Vital signs						
HR, beats/min	89.37 ± 20.26	89.17 ± 19.52	89.53 ± 20.31	89.21 ± 20.11	89.56 ± 21.12	0.995
NBPS, mmHg	119.05 ± 23.72	116.86 ± 22.55	119.52 ± 23.34	119.65 ± 24.73	120.17 ± 24.11	0.032
NBPD, mmHg	70.78 ± 139.63	68.81 ± 17.62	68.30 ± 18.26	68.78 ± 38.32	77.21 ± 275.08	0.032
NBPM, mmHg	80.94 ± 24.89	80.98 ± 17.84	81.28 ± 17.90	80.32 ± 18.45	81.17 ± 38.68	0.495
RR, times/min	19.76 ± 6.43	18.86 ± 6.32	19.58 ± 6.22	19.92 ± 6.35	20.66 ± 6.69	**<0** **.** **001**
SPO2, %	96.59 ± 4.18	97.02 ± 4.24	96.88 ± 3.54	96.07 ± 4.54	96.39 ± 4.25	**<0** **.** **001**
Temperature, °F	98.05 ± 3.51	97.96 ± 5.14	98.22 ± 1.07	97.93 ± 4.50	98.07 ± 1.24	0.002
Laboratory						
RBC, 10^9^/L	2.91 ± 0.65	3.08 ± 0.64	2.99 ± 0.66	2.90 ± 0.63	2.68 ± 0.58	**<0** **.** **001**
WBC, 10^9^/L	18.57 ± 8.09	16.96 ± 7.57	18.32 ± 7.16	18.64 ± 7.47	20.35 ± 9.57	**<0** **.** **001**
PLT, 10^9^/L	143.57 ± 62.94	145.61 ± 58.98	148.60 ± 61.10	143.63 ± 65.19	136.44 ± 65.74	0.014
RDW, (%)	16.25 ± 2.26	15.25 ± 1.97	15.87 ± 2.05	16.47 ± 2.19	17.39 ± 2.23	**<0** **.** **001**
Hb, g/dl	8.64 ± 1.86	9.18 ± 1.86	8.89 ± 1.92	8.60 ± 1.80	7.89 ± 1.62	**<0** **.** **001**
Hct, mg/dl	37.07 ± 6.00	38.52 ± 5.77	37.82 ± 6.03	36.69 ± 5.85	35.28 ± 5.86	**<0** **.** **001**
BUN, mg/dl	45.59 ± 25.94	20.77 ± 5.09	32.70 ± 5.97	47.69 ± 9.82	81.08 ± 21.99	**<0** **.** **001**
Albumin (g/dl)	3.34 ± 0.58	3.63 ± 0.54	3.43 ± 0.53	3.26 ± 0.53	3.04 ± 0.53	**<0** **.** **001**
Lactic acid	2.77 ± 1.54	2.57 ± 1.32	2.66 ± 1.43	2.83 ± 1.59	3.03 ± 1.74	0.001
Glucose (mg/dl)	202.78 ± 70.64	171.84 ± 54.18	192.07 ± 63.53	210.53 ± 71.75	236.58 ± 74.72	**<0** **.** **001**
Creatinine (mg/dl)	1.29 ± 0.62	0.88 ± 0.28	1.11 ± 0.42	1.39 ± 0.57	1.79 ± 0.69	**<0** **.** **001**
Lymhocytes	13.36 ± 6.83	15.61 ± 7.08	13.82 ± 6.48	12.83 ± 6.83	11.20 ± 6.16	**<0** **.** **001**
Monocyte	6.69 ± 3.36	6.23 ± 3.20	6.38 ± 3.25	6.88 ± 3.39	7.28 ± 3.50	**<0** **.** **001**
Neutrohil	82.48 ± 7.93	79.80 ± 8.03	81.82 ± 7.90	83.42 ± 7.69	84.86 ± 7.21	**<0** **.** **001**
Comorbidities						
HF						0.088
HFmrEF	936 (39.28%)	224 (41.30%)	256 (37.94%)	246 (35.53%)	210 (42.34%)	
HFpEF	901 (34.36%)	221 (33.73%)	210 (34.81%)	209 (37.09%)	261 (31.82%)	
HFrEF	633 (26.36%)	162 (24.97%)	157 (27.25%)	158 (27.37%)	156 (25.84%)	
AF	801 (24.02%)	187 (22.45%)	199 (23.89%)	206 (24.73%)	209 (25%)	0.614
HTN	604 (24.45%)	176 (28.53%)	182 (29.50%)	142 (23.01%)	104 (16.80%)	**<0** **.** **001**
PNA	922 (37.33%)	156 (25.28%)	215 (34.85%)	251 (40.68%)	300 (48.47%)	**<0** **.** **001**
CVA	257 (10.40%)	48 (7.78%)	68 (11.02%)	70 (11.35%)	71 (11.47%)	0.104
T2DM	931 (37.69%)	160 (25.93%)	215 (34.85%)	252 (40.84%)	304 (49.11%)	**<0** **.** **001**
T1DM	32 (1.30%)	5 (0.81%)	3 (0.49%)	11 (1.78%)	13 (2.10%)	0.035
HLD	1,210 (48.99%)	292 (47.33%)	340 (55.11%)	297 (48.14%)	281 (45.40%)	**0** **.** **004**
MI	522 (21.13%)	122 (19.77%)	138 (22.37%)	131 (21.23%)	131 (21.16%)	0.741
CAD	785 (31.78%)	66 (10.70%)	148 (23.99%)	225 (36.47%)	346 (55.90%)	**<0** **.** **001**
COPD	567 (22.96%)	118 (19.12%)	131 (21.23%)	158 (25.61%)	160 (25.85%)	0.009
Severity scores						
SOFA	4.61 ± 2.60	4.01 ± 2.47	4.48 ± 2.59	4.66 ± 2.48	5.27 ± 2.71	<0.001
APSIII	42.26 ± 15.78	35.58 ± 14.53	39.86 ± 14.68	43.18 ± 14.01	50.36 ± 16.01	<0.001
SAPSII	37.06 ± 10.96	31.96 ± 10.14	35.66 ± 10.42	38.13 ± 10.18	42.45 ± 10.37	<0.001
OASIS	30.76 ± 7.71	28.92 ± 7.46	30.55 ± 7.56	31.19 ± 7.84	32.39 ± 7.55	<0.001
APACHEII	13.56 ± 2.90	13.56 ± 3.03	13.50 ± 3.10	13.68 ± 2.61	13.50 ± 2.83	0.081
Drugs						
ACEI/ARB	774 (31.34%)	206 (33.39%)	227 (36.79%)	200 (32.41%)	141 (22.78%)	**<0** **.** **001**
Stains	519 (21.01%)	112 (18.15%)	141 (22.85%)	121 (19.61%)	145 (23.42%)	0.066
Diuretics	958 (38.79%)	274 (44.41%)	263 (42.63%)	246 (39.87%)	175 (28.27%)	**<0** **.** **001**
Anticoagulants	2,297 (93%)	557 (90.28%)	570 (92.38%)	586 (94.98%)	584 (94.35%)	0.005
Antilatelet	570 (23.08%)	110 (17.83%)	149 (24.15%)	169 (27.39%)	142 (22.94%)	**<0** **.** **001**
Treatment						
Ventilation	1,198 (48.50%)	285 (46.19%)	290 (47%)	291 (47.16%)	332 (53.63%)	0.031
Outcomes						
30-day mortality	362 (14.66%)	25 (4.05%)	52 (8.43%)	104 (16.86%)	181 (29.24%)	**<0** **.** **001**
In-hospital mortality	87 (3.52%)	3 (0.49%)	11 (1.78%)	25 (4.05%)	48 (7.75%)	**<0** **.** **001**
365-day mortality	378 (15.30%)	25 (4.05%)	55 (8.91%)	108 (17.50%)	190 (30.69%)	**<0** **.** **001**
ICU mortality	271 (10.97%)	22 (3.57%)	40 (6.48%)	77 (12.48%)	132 (21.32%)	**<0** **.** **001**

Bold text indicates statistical significance.

Q1, 1st quartile; Q2, 2st quartile; Q3, 3st quartile; Q4, 4st quartile.

### Clinical outcomes

**Figure 2:**
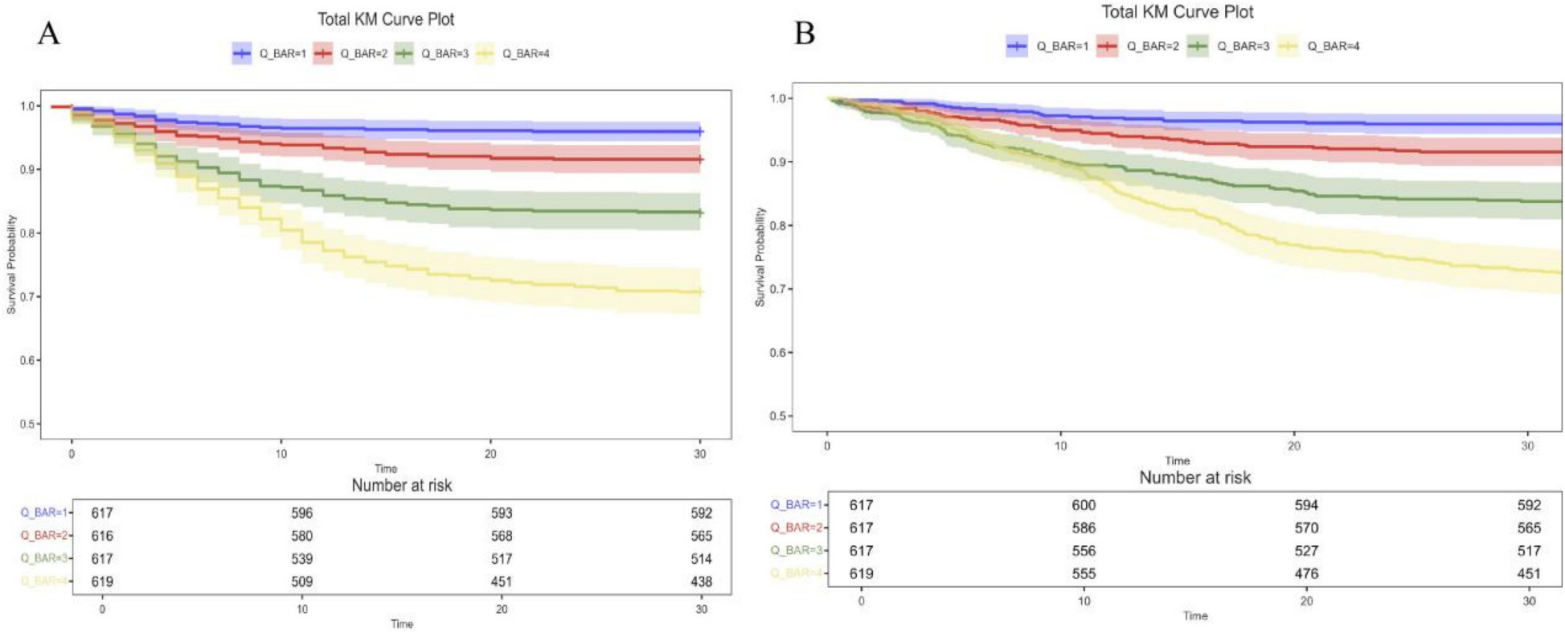
Kaplan–Meier survival analysis curves for all-cause mortality. Kaplan–Meier curves and cumulative incidence of 30-day **(A)** and 365-day **(B)** all-cause mortality stratified by BAR index. BAR, blood urea nitrogen to serum albumin ratio.

To investigate the independent effect of the BAR index on mortality, three Cox regression models were applied Table 3. Supplementary Table S1 shows the variance inflation factors, indicating that there is no multicollinearity between the variables. After adjusting for age, sex, and weight (Model 2), the HRs and 95% CIs for 30-day all-cause mortality for the BAR index categories (Q1, Q2, Q3, and Q4) were 1 (reference), 1.92 (1.18, 3.098), 3.92 (2.52–6.10), and 7.19 (0.98–0.99). Subsequent adjustments for age, body weight, PNA, PLT, RDW, neutrophils, hematocrit, blood urea nitrogen, lactate, lymphocytes, monocytes, diuretics, APSIII, SAPAII, and OASIS (Model 3) resulted in the following risk ratios for 30-day all-cause mortality: respectively:1.00 (reference), 1.52 (0.94–2.47), 2.28 (1.41–4.54) and 2.54 (1.42–4.54). In Model 2, the risk ratios for 365-day all-cause mortality were 1.00 (reference), 2.02 (95% CI: 1.26–3.26), 4.09 (95% CI: 2.64–6.35), and 7.49 (95% CI: 4.91–11.44), respectively. In model 3, the HRs were 1.00 (reference), 1.63 (95% CI: 1.01–2.64), 2.46 (95% CI: 1.53–3.95) and 2.75 (95% CI: 1.55–4.89). The results of the study showed that patients with a BAR index of 9.39 or higher had a significantly higher risk of all-cause mortality at both 30 and 365 days compared to those in Q2. The Kaplan–Meier survival curves illustrate the survival differences in 30- and 365-day mortality between the 4 BAR groups (Figure 2). Patients in the highest BAR index group (Q4) had significantly lower survival at 30 and 365 days than those in the lowest BAR index group (Q1) (log-rank *P* < 0.05).

**Table 3:** Logistic regression analysis of 30-day, 1-year, in-hospital, and ICU mortality in heart failure patients.

Exposure	Model 1		Model 2		Model 3	*P*-value
	HR (95% CI)	*p*-value	HR (95% CI)	*p*-value	HR (95% CI)	
30-day mortality						
BAR index	1.05 (1.05, 1.07)	<0.00001	1.05 (1.05, 1.07)	<0.00001	1.02 (1, 1.04)	0.04
Q1	Ref		Ref		Ref	
Q2	2.13 (1.32, 3.43)	0.002	1.92 (1.18, 3.098)	0.008	1.52 (0.94, 2.47)	0.09
Q3	4.48 (2.89, 6.93)	<0.00001	3.92 (2.52, 6.10)	<0.00001	2.28 (1.41, 3.66)	0.001
Q4	8.15 (5.37, 12.39)	<0.00001	7.19 (0.98, 0.99)	<0.00001	2.54 (1.42, 4.54)	0.002
*P* for trend	<0.00001		<0.00001		0.001	
365-day mortality						
BAR index	1.06 (1.05, 1.08)	<0.00001	1.06 (1.04, 1.08)	<0.00001	1.01 (0.96, 1.07)	0.65
Q1	Ref		Ref		Ref	
Q2	2.26 (1.41, 3.63)	0.001	2.02 (1.26, 3.26)	0.004	1.63 (1.01, 2.64)	0.08
Q3	4.68 (3.03, 7.22)	<0.00001	4.09 (2.64, 6.35)	<0.00001	2.46 (1.53, 3.95)	<0.00001
Q4	8.57 (5.65, 12.99)	<0.00001	7.49 (4.91, 11.44)	<0.00001	2.75 (1.55, 4.89)	0.001
*P* for trend	<0.00001		<0.00001		0.99	
In-hospital mortality						
BAR index	0.99 (0.97, 1.01)	0.32	0.99 (0.97, 1.01)	0.36	1.02 (0.06, 1.08)	0.56
Q1	Ref		Ref		Ref	
Q2	1.07 (0.30, 3.84)	0.91	1.05 (0.29, 3.78)	0.94	1.21 (0.32, 4.53)	0.78
Q3	1.46 (0.44, 4.84)	0.54	1.58 (0.47, 5.26)	0.46	3.28 (0.91, 11.74)	0.06
Q4	11.33 (0.35, 3.65)	0.83	1.15 (0.36, 3.74)	0.81	3.89 (0.92, 16.34)	0.06
*P* for trend	0.98		0.92		0.01	
ICU mortality						
BAR index	0.10 (0.98, 1.01)	0.38	0.99 (0.98, 1.01)	0.37	1.02 (0.10, 1.05)	0.14
Q1	Ref		Ref		Ref	
Q2	0.60 (0.36, 1.02)	0.05	0.59 (0.35, 1.00)	0.05	0.60 (0.35, 1.03)	0.06
Q3	0.70 (0.44, 1.13)	0.15	0.68 (0.42, 1.10)	0.12	0.61 (0.36, 1.04)	0.07
Q4	0.56 (0.35, 0.87)	0.01	0.54 (0.33, 0.85)	0.01	0.49 (0.26, 0.95)	0.03
*P* for trend	0.04		0.04		0.11	

Model 1: unadjusted.

Model 2: adjusted for age, sex and weight.

Model 3: adjusted for age, sex, weight, PNA, AKI, RDW, HCT, APSIII, SAPAII, OASIS, Neutrophils, Blood Urea Nitrogen (BUN), Lactate, Lymphocytes and Diuretics.

### Restricted cubic spline

**Figure 3:**
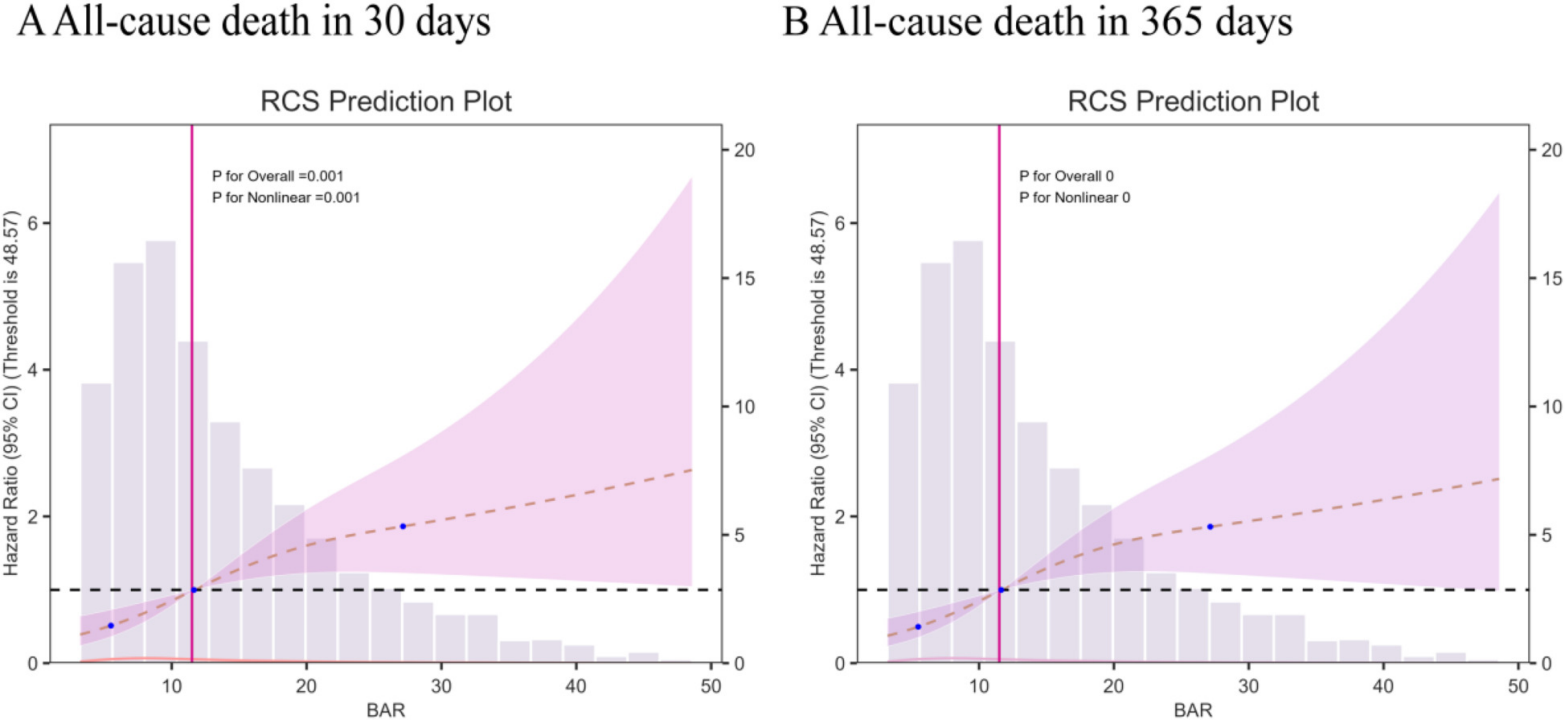
Multivariable RCS regression showed the nonlinear association between the BAR index and 30-day mortality **(A)** and one-year **(B)** mortality after full adjustment.

According to the multivariate RCS model, the risk of 30- and 365-day mortality was found to increase nonlinearly (*p*-value <0.05, non-linear *p* > 0.05) with increasing BAR index (Figure 3). In addition, a combination of Cox proportional risk modeling and two-stage Cox proportional risk modeling was used to investigate the nonlinear relationship between the level of BAR in heart failure patients and the aforementioned mortality rates (both *P* for log-likelihood ratio <0.05) (Supplementary Table S3). The inflection points for 30-day and 365-day all-cause mortality were 13.23 and 12.97, respectively. When the BAR index was below 13.23 or 12.97, the adjusted HR for 30- and 365-day mortality increased by a factor of 1 for each unit increase in the BAR level (HR 1.14; 95% CI, 1.06–1.23; HR 1.15; 95% CI, 1.06–1.24, respectively). However, when the BAR was greater than the inflection point, there was no significant association with 30- and 365-day mortality.

### Subgroup analysis

**Figure 4:**
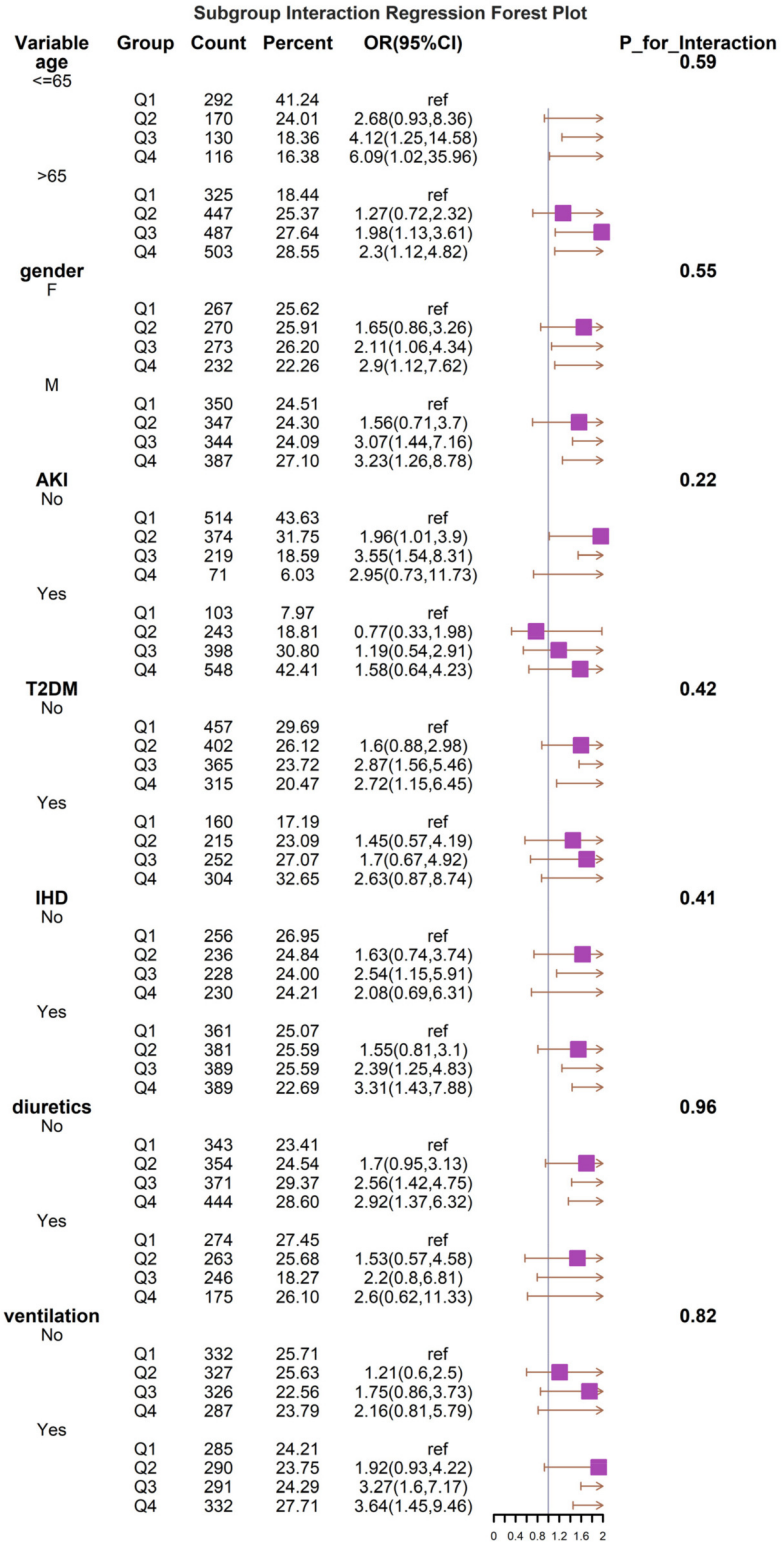
Forest plots of stratified analyses of BAR index and all-cause mortality (30 days). With the exception of the stratified variable itself, the adjustment approach is the same as for Model 3.

The results show a subgroup analysis of 30-day all-cause mortality (Figure 4). In subgroups defined by age, sex, T2DM, AKI, CAD, diuretics, and mechanical ventilation status, Q4 consistently demonstrated a higher risk of death, with or without adjustment for covariates. The results of the subgroup analyses indicated that the BAR index was similarly associated with the 30-day risk of death in patients with critical heart failure in most subgroups of the population. The model was not significant for the interaction test of covariates with the BAR index.

### Establishment and validation of the prediction model

**Figure 5:**
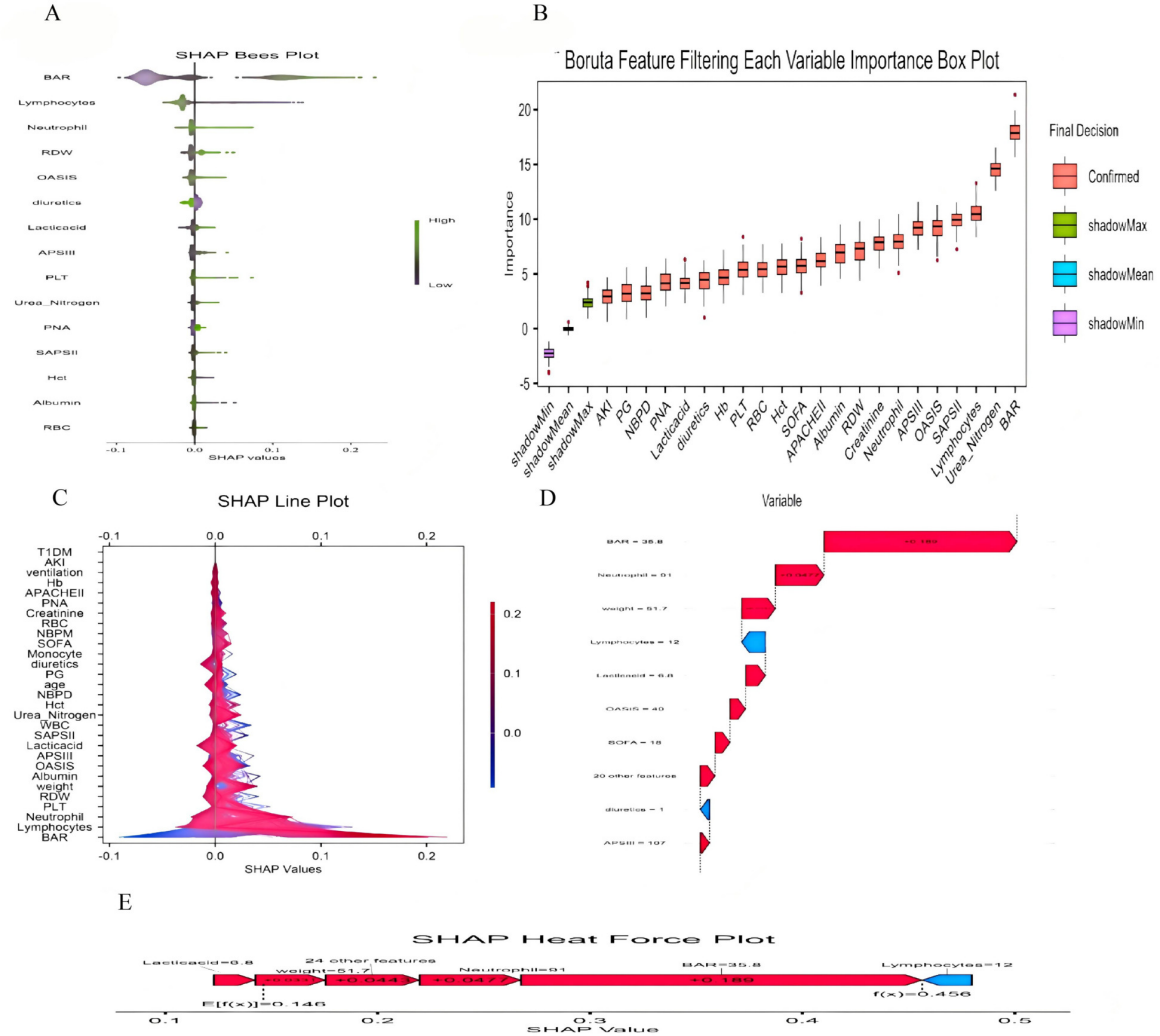
**(A)** SHAP Bees Plot.The distribution of each feature's impact on the model output. Each point represents a patient in a row. The color of the points indicates the feature value: purple represents higher values, while green represents lower values. **(B)** Feature Selection Using the Boruta Algorithm to Analyze the Relationship Between Various BAR Indices and 30-day Mortality. The x-axis displays the names of each variable, while the y-axis represents the Z-values of each variable. Box plots illustrate the Z-values of each variable calculated in the model, where red boxes indicate important variables, green boxes indicate tentative attributes and purple boxes indicate unimportant variables. **(C)** SHAP double-coordinate line graph. The SHAP plot shows the contribution of different features to the model prediction. The X-axis represents the SHAP value, and the Y-axis lists the names of each feature. The color bars indicate the magnitude of the feature values, with red representing higher feature values and blue representing lower feature values. **(D)** SHAP waterfall plot. The SHAP waterfall plot shows the composition of the predicted value for a single sample. The plot lists the contribution of each feature to the final predicted value. A red upward arrow indicates that the feature value increases the predicted value, while a blue downward arrow indicates that the feature value decreases the predicted value. **(E)** SHAP Heat Force plot.The SHAP heat force plot shows the contribution of each feature in the model prediction process. The arrows in the figure indicate the direction and magnitude of the feature's impact on the predicted value, with red arrows indicating an increase in the predicted value and blue arrows indicating a decrease in the predicted value.

**Figure 6:**
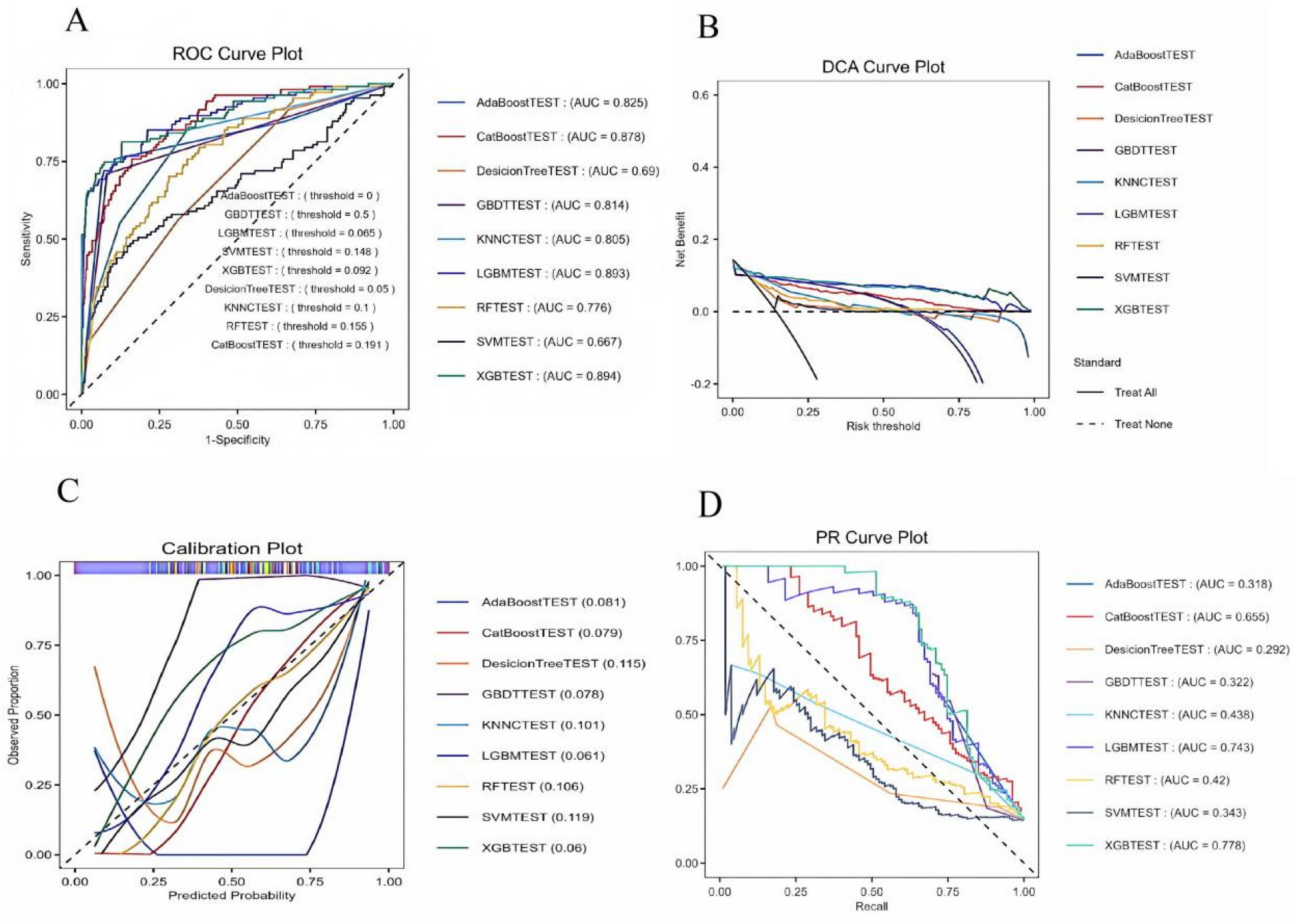
**(A)** ROC curves for the machine learning models. **(B)** DCA curves for the machine learning models. **(C)** Callibration for the machine learning models. **(D)** PR curves for the machine learning models. DT, decision tree; RF, random forest; XGB, extreme gradient boosting; LGB, light gradient boosting machine; KNNC, K-nearest neighbors classifier; SVM, support vector machine; Adaboost, adaptive boosting; Catboost, gradient boosting based; GBDT, gradient boosting machine; ROC, receiver operating characteristic; AUC, area under the curve.

We constructed 9 models to predict 30-day mortality in patients with heart failure, and Figure 5 shows the discriminative performance of these 9 models on the ROC curve. In addition, we evaluated and compared the performance of the models using metrics such as precision, accuracy, recall, and F1 score. Among the 9 base models, the XGBoost model demonstrated the best predictive performance with an AUC of 0.894 (95% CI: 0.85–0.93) for the XGBoost model. Following closely behind, the LGBM model showed considerable efficacy with an AUC of 0.893 (95% CI: 0.855–0.931), outperforming the remaining algorithms. The remaining models, while still showing good predictive power, are as follows in descending order of performance: CatBoost (AUC = 0.877, 95% CI: 0.842–0.914), KNNC (AUC = 0.805, 95% CI: 0.761–0.849), GBDT (AUC = 0.825, 95% CI: 0.773–0.877), AdaBoost (AUC = 0.814, 95% CI: 0.77–0.859), RF (AUC = 0.775, 95% CI: 0.729–0.823), DecisionTree (AUC = 0.689, 95% CI: 0.64–0.739), and SVM (AUC = 0.667, 95% CI: 0.602–0.731). According to the DCA curves (Figure 6B), each model showed a large net gain, indicating robust clinical validity of the models developed. Table 4 shows the detailed performance metrics of the nine models. XGBoost demonstrated the most balanced and excellent performance (precision = 0.813, recall = 0.654), LightGBM was the close alternative, and both were significantly better than the other models.

**Table 4:** Predictive performances of the nine machine learning models for predicting 30day mortality.

Models	Specificity	Precision	Accuracy	Recall	F1 score
CatBoost					
Training cohort	0.985	0.816	0.897	0.3738	0.512
Testing cohort	0.9950	0.8	0.940	0.25	0.380
RF					
Training cohort	0.985	0.608	0.862	0.130	0.215
Testing cohort	0.998	0.875	0.935	0.145	0.250
KNNC					
Training cohort	0.955	0.540	0.862	0.308	0.392
Testing cohort	0.981	0.312	0.917	0.104	0.156
DesicionTree					
Training cohort	0.9747	0.529	0.858	0.168	0.255
Testing cohort	0.993	0.636	0.931	0.145	0.237
XGB					
Training cohort	0.968	0.780	0.924	0.663	0.717
Testing cohort	0.993	0.878	0.964	0.604	0.716
SVM					
Training cohort	1.0	1.0	0.856	0.0093	0.018
Testing cohort	1.0	1.0	0.928	0.0208	0.040
LGBM					
Training cohort	0.974	0.8139	0.928	0.654	0.725
Testing cohort	0.993	0.885	0.967	0.645	0.746
AdaBoost					
Training cohort	0.917	0.593	0.887	0.710	0.646
Testing cohort	0.971	0.638	0.946	0.625	0.631
GBDT					
Training cohort	0.917	0.596	0.889	0.719	0.652
Testing cohort	0.973	0.652	0.948	0.625	0.638
Mean_scores					
Training cohort	0.964	0.697	0.885	0.415	0.459
Testing cohort	0.988	0.742	0.942	0.351	0.422

### Performance comparison of the model on the external validation set

Despite the inherent differences in baseline characteristics between the two datasets, our model exhibits strong generalization. The externally validated ROC curve yields an AUC of 0.924 (95% CI: 0.88–0.96).

### Selection of characteristic variable

The SHAP method is a comprehensive interpretation method applicable to both overall and individual sample analyses for interpreting models. For the overall interpretation, SHAP implies assessing the contribution of features to the model, with the five most important features (BAR, neutrophil count, weight, uric acid, and OASIS score) listed in descending order of importance (Figure 5).

## Discussion

This study is the first retrospective study to explore the association between BAR levels and all-cause mortality in critically ill patients with heart failure based on the MIMIC-IV database. Blood urea nitrogen/albumin ratio (BAR) is a newly discovered biological indicator in recent years that has the advantages of being noninvasive, easy to obtain, and widely used. Our study demonstrated a significant association between higher levels of the BAR index and increased 30-day and one-year mortality in critically ill patients with heart failure. This association held even after accounting for potential confounders, demonstrating the robustness of the findings. The study also found that patients' 30-day and one-year mortality rates were nonlinearly correlated with the BAR index.

In recent years, several clinical studies have demonstrated a correlation between elevated BUN levels and decreased ALB and poor prognosis in patients with cardiovascular disease, including acute coronary syndrome (14) and acute myocardial infarction (15). Blood urea nitrogen and albumin have long been of interest as single indicators, and the BAR index, as a combination of the two, with elevated levels that may reflect the patient's nutritional status, hepatic and renal function, as well as the inflammatory response, makes up for the lack of predictive performance of albumin or BUN alone and has the potential to be a complementary predictor for clinicians managing patients with heart failure. Lin ZB et al. (5) found that higher levels of BAR were associated with increased short-term mortality in heart failure patients undergoing intensive care. Zhao L et al. (16) conducted a cohort study with a median follow-up of 22 months and found that BAR was still associated with an increased risk of death in heart failure patients. A previous study on the association between BAR and heart failure patients only explored the relationship between BAR and in-hospital and 90-day mortality, and our study supports the previous studies; moreover, the present study reveals for the first time the relationship between the BAR index and 30-day and one-year mortality rates in heart failure patients, which is a major highlight of our study. In addition, we included patients from the U.S. Critical Care Database, which helps to provide more comprehensive results for clinical practice.

The exact biological mechanism between the BAR index and clinical outcome and prognosis in heart failure is not clear. In this study, we tried to understand the relationship between BAR and the poor prognosis of heart failure patients from the perspective of blood urea nitrogen and albumin, respectively. Blood urea nitrogen (BUN) is a major indicator of renal function, and elevated BUN levels suggest impaired renal function, blood volume deficiency, or neurohormonal activation, which may lead to poor prognosis in patients with heart failure through oxidative stress, activation of the sympathetic nervous system (SNS) and renin-angiotensin aldosterone system (RAAS), and activation of the renin-angiotensin aldosterone system (RAAS). Angiotensin aldosterone system (RAAS) leading to poor prognosis in patients with heart failure. In patients with heart failure, activation of RAAS promotes sodium retention and leads to reabsorption of blood urea nitrogen in the proximal renal tubules, whereas activation of SNS accelerates reabsorption of blood urea nitrogen in the distal renal tubules, and both mechanisms lead to elevated blood urea nitrogen (17). It is thus clear that activation of the SNS and RAAS systems is closely related to the progression of heart failure (18, 19).

Albumin is a protein synthesized by ALB from the liver and has a variety of biological functions such as antiplatelet aggregation, anticoagulation, maintenance of plasma osmolality, transport of a variety of substances, and inhibition of inflammatory responses (20). Previous studies have found a correlation between low albumin levels and poor prognosis in patients with heart failure (21). Heart failure is a disease of insufficient perfusion of relative organs due to systolic and/or diastolic dysfunction of the heart. Heart failure itself does not lead to hypoproteinemia, but patients with prolonged heart failure are often combined with infections, malnutrition, and other complications, which exacerbate the loss of albumin, and the reduction of albumin levels is closely related to inflammation, oxidative stress, endothelial dysfunction, and other pathologies, which can lead to circulating blood volume deficiency, disrupting fluid balance, and leading to heart failure decompensation.

Boruta's algorithm, a widely used method in feature selection, determines which features are most important for predicting the target variable by modeling randomness (22). The feature selection results of Boruta's algorithm in this study show that BAR significantly occupies the red region and exhibits high Z-scores in feature selection, suggesting that the BAR index may play a key role in this study, showing a significant association with the study objectives. However, we also recognize that this does not mean it is a decisive factor. This is because the Boruta algorithm may also be affected by correlations between data features. The results of multivariate COX regression analyses support the correlation between the BAR index and the risk of 30-day and 1-year mortality in patients with heart failure, which is consistent with the Boruta algorithm. In the subgroup analysis, regarding 30-day mortality, we observed no statistically significant results after stratification by variables such as age and gender. This may be due to the reduced sample size after stratification, resulting in a reduced effect size. However, the consistent direction of all results suggests the stability and reliability of the core results. Therefore, we believe that BAR can be used as a predictor of 30-day all-cause mortality in patients with heart failure.

With the rapid development of artificial intelligence in recent years, ML algorithms have been widely adopted in medical research, especially in predicting treatment outcomes and patient prognosis. We incorporate feature-significant variables into nine machine learning algorithms. The results show that the XGBoost algorithm exhibits strong performance in differentiation and calibration and shows significant net gains in clinical practice. Compared with traditional regression algorithms, the XGBoost model can automatically deal with nonlinear relationships between features, and through regularization and pruning techniques, XGBoost can reduce the risk of overfitting, thus improving the generalization ability of the model (23). In addition, the XGBoost model is robust to outliers and noisy data, and it reduces the effect of outliers by weighting the loss function, thus improving the stability of the model. This study also has some limitations. First, the present study was retrospective and could not establish a clear causal relationship between BAR index and heart failure. Nonetheless, a series of rigorous statistical methods were adopted to ensure that the results were robust and credible. Second, the BAR index is not monitored dynamically, and the BAR index obtained from taking the most severe urea nitrogen and albumin measurements may not be fully representative of the pathology in the body. Third, this is an observational study, and there may be some unmeasured or residual confounding effects that may affect the results. Fourth, when patients receive enteral or parenteral nutrition, it may affect urea nitrogen and albumin levels, potentially leading to an elevated BAR index. However, this effect may be mitigated due to the large sample size included in our study. Fifth, due to the inherent limitations of the database, the absence of some key metrics, such as NT-proBNP, may affect the comprehensiveness of our prediction model. Finally, although we used MIMIC-III as an external validation, the data were from a single center, and further large-scale, multicenter prospective studies are needed to validate the accuracy of our model.

## Conclusions

Our study demonstrated that the BAR index is a predictor of 30-day mortality and one-year mortality in heart failure ICU patients. In high-risk groups, the BAR may be a valuable tool for risk assessment and subsequent intervention, and further studies are needed in the future to validate the generalizability of the ratio and the optimal cut-off value and to determine the mechanisms underlying the association between the BAR and mortality in heart failure patients.

## Data Availability

The original contributions presented in the study are included in the article/Supplementary Material, further inquiries can be directed to the corresponding authors.
