# Supplementary material for: Correlation between blood urea nitrogen/albumin levels and 30-day all-cause mortality in critically Ill patients with heart failure: a retrospective cohort study and predictive model development based on machine learning
Source: Front Cardiovasc Med. 2025 Sep 5;12:1600640. doi: 10.3389/fcvm.2025.1600640 (PMC12446357; doi:10.3389/fcvm.2025.1600640)
Supplement: Supplementary file 1 [file Table1.pdf]

Supplementary Table1: Variance inflation factor between variables

Variable Name	GVIF	Df	GVIF^(1/(2*Df))
age	1.17434893039626	1	1.08367381180697
weight	1.20615340053353	1	1.09825015389643
PNA	1.11078198901457	1	1.05393642550895
PLT	1.19853118709148	1	1.09477449143259
RDW	1.53816279825135	1	1.24022691401668
Hct	1.40095940193823	1	1.18362130850126
UreaNitrogen	2.65002688505321	1	1.62789031726748
Q_BAR	3.21840988684997	3	1.21508565097508
Lacticacid	1.26372258965392	1	1.12415416631969
Lymphocytes	1.17125421127281	1	1.08224498671642
diuretics	1.13923501068723	1	1.06734952601631
APSIII	2.3577100979445	1	1.53548366905822
SAPSII	3.14125319616066	1	1.77235808914583
OASIS	2.02390895364762	1	1.42264154081329
